# Cytokine levels reflect tic symptoms more prominently during mild phases

**DOI:** 10.1186/s12868-023-00830-3

**Published:** 2023-10-31

**Authors:** SuHyuk Chi, Young Eun Mok, June Kang, Jeong-An Gim, Changsu Han, Moon-Soo Lee

**Affiliations:** 1 Department of Psychiatry, Korea University Guro Hospital, 148, Gurodong-ro, Guro-gu, Seoul, 08308 Republic of Korea; 2 Department of Brain and Cognitive Engineering, Korea University, Seoul, Republic of Korea; 3 Medical Science Research Center, Korea University Guro Hospital, Seoul, Republic of Korea

**Keywords:** Tic disorder, Tourette disorder, Cytokines, Neuroinflammation

## Abstract

Tic disorder is a neuropsychiatric condition that affects 3% of all children and can have a significant impact on their quality of life. Cytokines, interferons, interleukins, lymphokines, and tumor necrosis factors are involved in the neuroinflammatory circuitry of tic disorders. This study aimed to identify the cytokines involved in the pathogenesis of tic disorders. We enrolled 44 patients with tic disorder and 38 healthy controls. Patients were free of psychotropic medications for at least 3 weeks. Whole blood samples were analyzed using a Luminex® human cytokine multiplex assay kit. Patients were divided into groups with “mild tics” and “above moderate tics” based on Yale Global Tic Severity Scale (YGTSS) scores for comparison. The final analysis included 35 patients (28 male and 7 female) and 31 controls (20 male and 11 female). In the mild tic group, interleukin (IL)-12 p70 negatively correlated with motor tic scores. Granulocyte-macrophage colony-stimulating factor, IL-4, IL-8, and tumor necrosis factor (TNF)-α were positively correlated to phonic tic scores. IL-12 p40 and TNF-α were positively correlated to total tic scores. IL-12 p70 and IL-17a negatively correlated to impairment scores and total YGTSS scores. Tic disorder patients and healthy controls exhibit different cytokine profiles. Only patients with mild symptoms exhibit significant correlations, suggesting that the correlations between cytokine levels and tic symptoms are more relevant during the mild or remission phases. Our results present the importance of IL-1β and TNF-α, among others, but the identification of key cytokines are still necessary.

## Background

Tic disorder is a neuropsychiatric condition affecting almost 3% of all children [1]. It is characterized by sudden, brief, repetitive, and involuntary movements or sounds, called tics, that involve various body parts. Although tics are not inherently harmful, they can be distressing, especially if severe or unpredictable. Tics also interfere with activities of daily living. Furthermore, patients with tic disorders have significantly higher probabilities of co-occurring attention-deficit hyperactivity disorder (ADHD), obsessive-compulsive disorder (OCD), and depression than the general population, which can further complicate the difficulties they face. Multiple trials have attempted to identify efficacious treatment options for the disease [2].

Tic disorders typically begin during childhood and may last for less than a year or even an entire lifetime. Neither the exact cause nor the factors determining the disease duration are known, but they are believed to involve a combination of various genetic and environmental factors. Previous studies have supported the neuroinflammatory aspect of tic disorders. This idea was driven by the description of post-infection cases of tic disorders, mainly associated with group A β-hemolytic streptococci [3]. Other studies have described tic disorder cases that met the criteria for pediatric autoimmune neuropsychiatric disorders associated with streptococcus (PANDAS) or pediatric acute-onset neuropsychiatric syndrome (PANS) [4]. These findings have naturally led to research on the involvement of cytokines in tic disorder patients.

Cytokines are small proteins involved in various immune modulatory processes. Chemokines, interferons, interleukins, lymphokines, and tumor necrosis factors are considered subgroups of cytokines. Clinical research has revealed that they can be used as biomarkers for certain diseases or as treatment options for conditions such as hepatitis, multiple sclerosis, and some types of cancer [5]. Research on the association between cytokine levels and psychiatric disorders has yielded significant results. Adolescents with major depressive disorder show different cytokine profiles than those of healthy individuals, which are further altered by antidepressant treatment [6, 7]. A recent meta-analysis on adolescent depression patients concluded that tumor necrosis factor (TNF)-α is higher in patients than in controls, but their results failed to reach statistical significance [8]. Patients with bipolar disorder and obsessive-compulsive disorder have also shown different cytokine levels compared to those of controls in several studies [9, 10].

A comprehensive review by Martino et al. stated that studies on cytokines and immunoglobulins suggest an overactive immune response at the systemic level [11]. Unfortunately, studies on the association between individual cytokines and tic disorders have yielded diverse results [12]. This may be because tic disorders start at a young age and pediatric patients have difficulty participating in clinical research [13]. However, older patients usually exhibit milder symptoms due to the clinical characteristics of the disease, and thus may show different neuroinflammatory profiles than those of younger patients. Such difficulties might have resulted in the enrollment of heterogeneous participant groups, which in turn led to heterogeneous results.

As demonstrated, cytokines likely play a role in the pathogenesis or progression of tic disorders. However, further studies are requisite to substantiate these findings. Therefore, in the present study, we gathered a homogeneous group of young, medication-free patients with tic disorders using semi-structured interviews with high diagnostic accuracy. Blood cytokine levels were analyzed and compared with healthy controls to identify the cytokines involved in the neuroinflammatory circuitry of tic disorders. Through this approach we aim to identify potential biomarkers for predicting the pathogenesis and prognosis of tic disorder in the form of cytokines.

## Methods

A total of 44 patients with tic disorder and 38 healthy controls were enrolled. All participants were between the ages of 6 and 18 years; had IQ scores above 70, as measured by the Korean version of the Wechsler Intelligence Scale for Children (K-WISC-IV); were free of psychotropic medication for at least 3 weeks; and had no past medical history of neurologic disorders, head trauma, tumors, or seizures. Child and adolescent psychiatrists clinically diagnosed patients with tic disorders based on the 5th edition of the Diagnostic and Statistical Manual of Mental Disorders.

Patients were recruited from the Department of Psychiatry of Korea University Guro Hospital. The healthy controls were recruited from local schools and kindergartens. Patients were assessed using the Korean version of the Kiddie-Schedule for Affective Disorders and Schizophrenia-Present and Lifetime Version for psychiatric comorbidities and the Yale Global Tic Severity Scale (YGTSS) for tic disorder symptom severity. The YGTSS is a clinical rating instrument specifically applied in Tourette’s syndrome and tic disorders [14]. It encompasses the number, frequency, intensity, complexity, and interference of both motor and phonic symptoms by assessing tic symptoms using a combination of motor tic scores, phonic tic scores, and impairment rating scores. Whole blood samples of all participants were taken for analysis of cytokine levels using a Luminex® human cytokine multiplex assay kit. The cytokines for assessment were selected based on previous literature and sorted into pro-inflammatory and anti-inflammatory cytokines [4, 15–20].

Patients were divided into two subgroups based on YGTSS scores: scores below 20 were seen as patients with “mild tics,” and score of 20 or above were seen as patients with “above moderate tics” [21–23]. T-tests and one-way analysis of variance (ANOVA) with Bonferroni corrections were performed to compare cytokine levels in the patient, control, and patient subgroups. Correlation analysis between the YGTSS scores and cytokine levels was performed for all patients and their two subgroups.

Patients and guardians were asked about the time of initial symptom onset. The duration between the initial onset and hospital visit for enrollment in this study (duration until visit) was calculated in months. Correlation analysis was performed between cytokine levels and the duration until the visit.

Statistical analyses were performed using SPSS version 23 software (IBM Corp., Armonk, NY, USA). The significance level was set at p < 0.05. The study was approved by the Institutional Review Board (IRB) of Korea University Guro Hospital. Written consent was obtained from the parents or legal guardians of all participants.

## Results

### Demographic and clinical characteristics

Several participants were excluded from the analysis. Of the 44 patients, six withdrew their consent, and three patients failed to acquire blood samples. Of the 38 controls, two were reassigned to the patient group after the initial clinical assessment, four withdrew consent, and one failed to provide a blood sample. A total of 35 patients (28 male and 7 female) and 31 controls (20 male and 11 female) were included in the final analysis. Patients and controls showed significantly different intelligence quotients (94.80 ± 2.85 and 102.90 ± 10.68 respectively). Among the patients, 11 were diagnosed with comorbid ADHD, 1 with generalized anxiety disorder, and 1 with comorbid enuresis. Tables 1 and 2 summarizes the patients’ demographic and clinical characteristics.

**Table 1:** Demographic and clinical variables of patients and controls

	Patients (n = 35)	Controls (n = 31)	*p* value
Sex (male/female)	28/7	20/11	
Age (years)	9.40 ± 2.85	9.87 ± 2.32	0.468
IQ	94.80 ± 10.15	102.90 ± 10.68	0.002*
Duration until visit (months)	44.74 ± 37.60		
YGTSS score	24.57 ± 13.32		
Motor tic score	7.03 ± 3.89		
Phonic tic score	4.69 ± 4.63		
Total tic score	11.71 ± 6.41		
Impairment score	12.86 ± 7.89		
Comorbidities (male/female)			
ADHD	9/2		
Generalized anxiety disorder	1/0		
Enuresis	1/0		

*p < 0.05

*IQ* intelligence quotient, *YGTSS* Yale Global Tic Severity Scale, *ADHD* attention-deficit hyperactivity disorder

**Table 2:** Demographic and clinical variables of patient sub-groups

	Mild (n = 13)	Above moderate (n = 22)	*p* value
Sex (male/female)	10/3	18/4	
Age (years)	7.46 ± 1.61	10.55 ± 2.82	0.001*
IQ	90.77 ± 10.787	97.18 ± 9.19	0.070
Duration until visit (months)	22.50 ± 16.90	56.86 ± 40.44	0.002*
YGTSS score	14.46 ± 6.62	30.55 ± 12.70	> 0.001*
Motor tic score	4.85 ± 3.72	8.32 ± 3.46	0.009*
Phonic tic score	1.15 ± 2.82	6.77 ± 4.23	> 0.001*
Total tic score	6.00 ± 3.11	15.09 ± 5.37	> 0.001*
Impairment score	8.46 ± 3.76	15.45 ± 8.58	0.002*
Comorbidities (male/female)			
ADHD	5/1	4/1	
Generalized anxiety disorder	1/0	0/0	
Enuresis	0/0	1/0	

*p < 0.05

*IQ* intelligence quotient, *YGTSS* Yale Global Tic Severity Scale, *ADHD* attention-deficit hyperactivity disorder

### Cytokine level differences between patients and controls

**Fig. 1:**
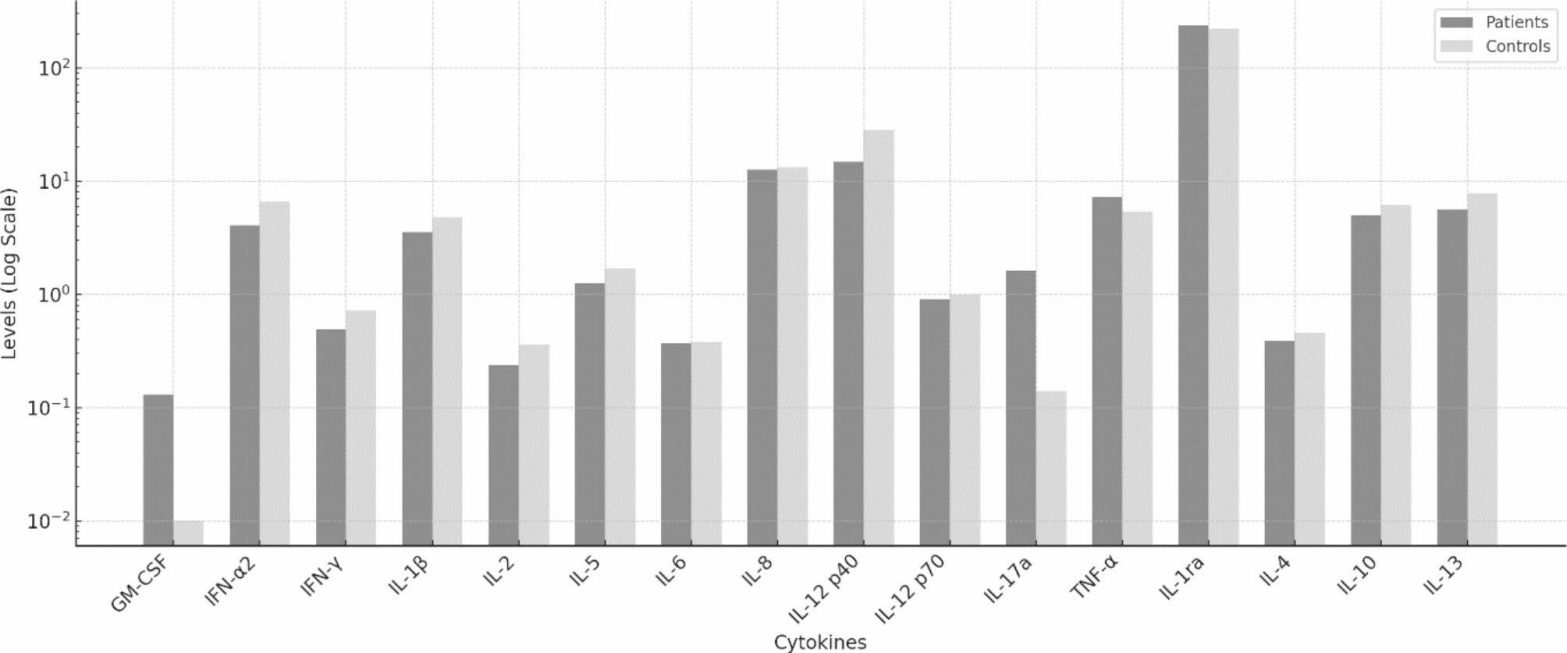
Cytokine levels of all patients and controls visualized using a logarithmic scale

**Fig. 2:**
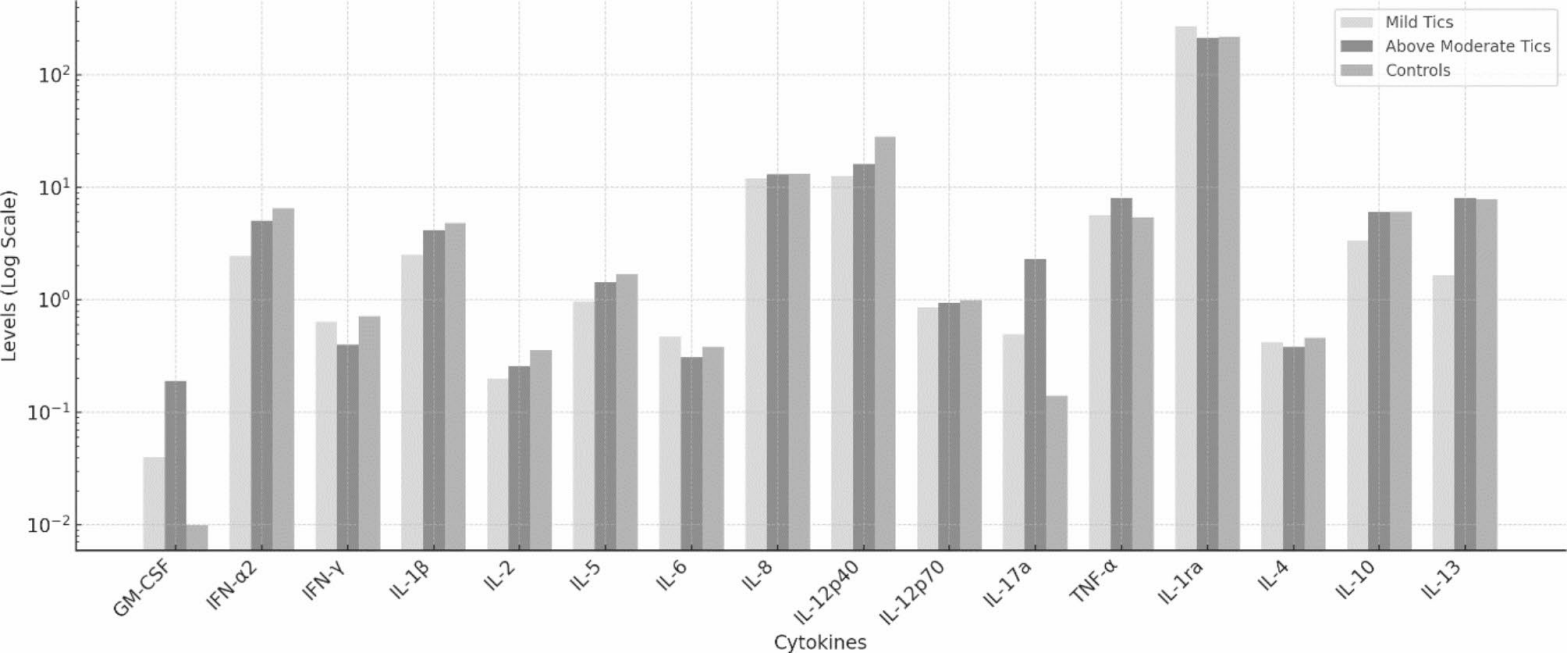
Cytokine levels of patient subgroups and controls visualized using a logarithmic scale

A t-test analysis of cytokine levels between patients and controls revealed no significant differences. The results are summarized in Table 3 and visualized in Fig. 1. ANOVA with according post-hoc analyses comparing mild, above moderate tic patients with controls revealed that interleukin (IL)-1β levels were significantly lower in mild tic patients compared to healthy controls (2.51 ± 1.49 and 4.81 ± 3.02, respectively). Table 4 presents the ANOVA results and Fig. 2 shows a graphical representation of the data.

**Table 3:** T-test analysis of cytokine levels between patients and controls

Cytokine	Patients	Controls	*p* value
*Pro-inflammatory*			
GM-CSF	0.13 ± 0.44	0.01 ± 0.05	0.111
IFN-α2	4.09 ± 6.98	6.57 ± 17.05	0.433
IFN-γ	0.49 ± 0.52	0.72 ± 0.68	0.112
IL-1β	3.54 ± 2.57	4.81 ± 3.02	0.070
IL-2	0.24 ± 0.16	0.36 ± 0.49	0.153
IL-5	1.26 ± 0.96	1.69 ± 1.27	0.117
IL-6	0.37 ± 0.24	0.38 ± 0.15	0.898
IL-8	12.65 ± 6.77	13.18 ± 5.56	0.729
IL-12 p40	14.79 ± 9.93	28.21 ± 59.35	0.192
IL-12 p70	0.91 ± 0.43	0.99 ± 0.23	0.318
IL-17a	1.63 ± 6.15	0.14 ± 0.17	0.160
TNF-α	7.18 ± 10.14	5.40 ± 1.78	0.339
*Anti-inflammatory*			
IL-1ra	235.21 ± 158.40	219.95 ± 105.13	0.651
IL-4	0.39 ± 0.23	0.46 ± 0.20	0.204
IL-10	4.98 ± 4.11	6.09 ± 5.09	0.337
IL-13	5.67 ± 8.60	7.76 ± 9.63	0.356

*p < 0.05

*GM-CSF* granulocyte-macrophage colony-stimulating factor; *IFN* interferon, *IL* interleukin, *TNF* tumor necrosis factor

**Table 4:** One-way analysis of variance comparing the cytokine levels of the mild and above moderate tic patients and those of the controls

Cytokine	Group	mean	SD	F-value	*p* value
*Pro-inflammatory*					
GM-CSF	Mild tics	0.04	0.10	2.12	0.128
	Above moderate tics	0.19	0.55		
	Controls	0.01	0.05		
IFN-α2	Mild tics	2.45	2.48	0.48	0.623
	Above moderate tics	5.05	8.53		
	Controls	6.57	17.05		
IFN-γ	Mild tics	0.64	0.69	1.98	0.147
	Above moderate tics	0.40	0.39		
	Controls	0.72	0.68		
IL-1β	Mild tics	2.51	1.49	3.19	0.048*
	Above moderate tics	4.15	2.90		
	Controls	4.81	3.02		
IL-2	Mild tics	0.20	0.10	1.16	0.322
	Above moderate tics	0.26	0.18		
	Controls	0.36	0.49		
IL-5	Mild tics	0.97	0.66	1.95	0.150
	Above moderate tics	1.43	1.08		
	Controls	1.69	1.27		
IL-6	Mild tics	0.47	0.31	2.58	0.084
	Above moderate tics	0.31	0.15		
	Controls	0.38	0.15		
IL-8	Mild tics	12.02	4.13	0.16	0.849
	Above moderate tics	13.02	8.00		
	Controls	13.18	5.56		
IL-12p40	Mild tics	12.55	5.59	0.89	0.417
	Above moderate tics	16.11	11.70		
	Controls	28.21	59.35		
IL-12p70	Mild tics	0.85	0.31	0.79	0.459
	Above moderate tics	0.94	0.49		
	Controls	0.99	0.23		
IL-17a	Mild tics	0.49	1.42	1.60	0.211
	Above moderate tics	2.31	7.67		
	Controls	0.14	0.17		
TNF-α	Mild tics	5.64	2.19	0.90	0.410
	Above moderate tics	8.10	12.71		
	Controls	5.40	1.78		
*Anti-inflammatory*					
IL-1ra	Mild tics	271.61	196.50	0.85	0.432
	Above moderate tics	213.71	131.36		
	Controls	219.95	105.13		
IL-4	Mild tics	0.42	0.29	0.96	0.390
	Above moderate tics	0.38	0.19		
	Controls	0.46	0.20		
IL-10	Mild tics	3.34	0.96	1.85	0.165
	Above moderate tics	6.00	4.96		
	Controls	6.09	5.09		
IL-13	Mild tics	1.67	0.81	2.56	0.086
	Above moderate tics	8.04	10.18		
	Controls	7.76	9.63		

*p < 0.05

### Correlation analyses

Correlation analyses between cytokine levels and YGTSS scores for all patients and the moderate-tics subgroup revealed no significant correlations. However, several significant correlations were observed in the mild tics subgroup. IL-12 p70 levels negatively correlated with motor tic scores. Granulocyte-macrophage colony-stimulating factor, IL-4, IL-8, and TNF-α levels positively correlated with phonic tic scores. IL-12 p40 and TNF-α levels positively correlated with total tic scores. IL-12, p70, and IL-17a le7vels were negatively correlated with impairment and total YGTSS scores. Table 5 presents the results of the correlation analyses.

**Table 5:** Correlation analysis between the cytokine levels and YGTSS scores of the tic disorder patient subgroups

Tic subgroup	Cytokine	Motor tic score	Phonic tic score	Total tic score	Impairment score	YGTSS score
All patients	GM-CSF	-0.075	0.075	0.009	0.082	0.053
	IFN-α2	0.065	0.089	0.103	0.137	0.131
	IFN-γ	-0.320	-0.167	-0.315	-0.295	-0.327
	IL-1β	0.014	0.092	0.075	0.108	0.100
	IL-2	0.116	0.087	0.134	0.141	0.148
	IL-5	0.112	0.037	0.095	0.066	0.085
	IL-6	-0.317	-0.079	-0.250	-0.151	-0.210
	IL-8	0.112	-0.119	-0.018	0.068	0.032
	IL-12 p40	0.081	0.007	0.054	-0.027	0.010
	IL-12 p70	-0.056	-0.079	-0.091	-0.175	-0.148
	IL-17a	-0.026	0.073	0.037	0.110	0.083
	TNF-α	-0.154	0.179	0.036	-0.040	-0.006
	IL-1ra	-0.083	-0.006	-0.055	0.007	-0.022
	IL-4	-0.238	0.053	-0.106	-0.097	-0.108
	IL-10	0.087	0.146	0.159	0.114	0.143
	IL-13	0.063	0.072	0.090	0.181	0.151
Mild tics	GM-CSF	-0.344	0.946^**^	0.296	0.175	0.239
	IFN-α2	0.353	0.024	0.407	0.315	0.370
	IFN-γ	-0.234	0.089	-0.195	-0.440	-0.341
	IL-1β	-0.070	0.228	0.085	-0.037	0.019
	IL-2	0.254	0.072	0.332	0.269	0.309
	IL-5	0.405	-0.147	0.343	0.175	0.261
	IL-6	-0.070	0.104	-0.003	0.167	0.093
	IL-8	-0.042	0.745^**^	0.486	0.557	0.545
	IL-12 p40	0.271	0.414	0.595^*^	0.469	0.546
	IL-12 p70	-0.682^*^	0.321	-0.524	-0.583^*^	-0.577^*^
	IL-17a	-0.443	-0.099	-0.560	-0.664^*^	-0.640^*^
	TNF-α	0.202	0.586^*^	0.642^*^	0.650^*^	0.671^*^
	IL-1ra	0.181	-0.216	0.046	0.049	0.050
	IL-4	-0.294	0.915^**^	0.329	0.142	0.236
	IL-10	-0.188	-0.285	-0.411	-0.101	-0.251
	IL-13	0.012	0.421	0.315	0.297	0.317
Above moderate tics	GM-CSF	-0.192	-0.143	-0.250	-0.144	-0.206
	IFN-α2	0.020	-0.062	-0.035	-0.145	-0.116
	IFN-γ	-0.326	-0.004	-0.234	-0.153	-0.206
	IL-1β	-0.146	-0.113	-0.193	0.043	-0.052
	IL-2	-0.036	0.062	0.024	0.115	0.090
	IL-5	-0.143	0.009	-0.094	0.040	-0.012
	IL-6	-0.097	0.012	-0.060	-0.121	-0.110
	IL-8	0.119	-0.296	-0.151	0.058	-0.024
	IL-12 p40	-0.103	-0.178	-0.215	-0.232	-0.252
	IL-12 p70	0.004	-0.151	-0.118	-0.150	-0.154
	IL-17a	-0.014	-0.046	-0.047	-0.076	-0.073
	TNF-α	-0.317	0.154	-0.102	-0.134	-0.136
	IL-1ra	0.165	-0.056	0.073	0.096	0.098
	IL-4	-0.178	-0.158	-0.252	-0.145	-0.207
	IL-10	-0.068	0.018	-0.034	0.048	0.019
	IL-13	-0.108	-0.191	-0.229	0.047	-0.064

*p < 0.05, **p < 0.01

*YGTSS* Yale Global Tic Severity Scale, *GM-CSF* granulocyte-macrophage colony-stimulating factor, *IFN* interferon, *IL* interleukin, *TNF* tumor necrosis factor

Correlation analyses between cytokine levels and the duration until visit for all patients did not reveal any significant relationships. Only the interferon (IFN)-γ levels were significantly higher in patients with duration until visit shorter than or equal to 6 months (n = 5) than they were in patients with duration until visit longer than 6 months (0.98 ± 0.74 vs. 0.41 ± 0.45).

## Discussion

Our study strived to investigate the cytokine networks of tic disorder patients. The established literature recognizes the influence of psychotropic medication, particularly antipsychotics, on cytokine levels. We introduced a washout period to minimize such effects. The main regimens used to treat tic disorders in Korea are aripiprazole and risperidone. Aripiprazole has an elimination half-life of 75 h (94 h for its active metabolite), and risperidone has an elimination half-life of 22 h [24, 25]. Based on this information and past studies we considered three weeks to be sufficient for a washout period [26, 27]. Fortunately, only one patient with above moderate tics was taking 15 mg of aripiprazole prior to participation, which was stopped three weeks before assessments.

This study revealed that tic disorder patients and healthy controls show different cytokine profiles. Particularly, patients with mild tic symptoms showed lower IL-1β levels than those of controls. The exact pathway by which IL-1β may affect in tic disorder is also not well known, and past literature on the association between tic disorder and IL-1β show mixed results [15, 16]. However, some studies suggest that inflammatory cytokines including IL-1β may modulate tryptophan metabolism, leading to over-production of toxic metabolites, further leading to abnormal responsiveness to stress [28, 29]. Our study also showed that the YGTSS scores of mild tic patients correlated with the levels of various cytokines: GM-CSF, IL-4, IL-8, IL-12 p40, and TNF-α levels exhibited positive correlations, whereas IL-12 p70 and IL-17a levels exhibited negative correlations. Previous studies have investigated the relationship between tic disorders and cytokines to identify the cytokines that play key roles. A recent research by Tao et al. compared tic disorder patients with controls, determining that patients had higher levels of IL-6 while levels of IL-2, IL-4, IL10, TNF-α, and IFN-γ were lower than controls [18]. Leckman et al. also compared tic disorder patients and healthy controls, showing that the patients had elevated IL-12 and TNF-α levels [30]. A recent meta-analysis summarizing 25 studies also identified small-to-large effect sizes for increased IL-6 levels and a large effect size for increased TNF-α levels in tic disorder patients [31]. Additionally, although not directly about tic disorders, some studies found that OCD patients, which is comorbid with tic disorder, have increased monocytes compared to healthy controls, which in turn release more cytokines including GM-CSF, IL-1β, IL-6, IL-8, and TNF-α [10, 32]. Parker-Athill showed that TNF-α levels were associated with tic symptom exacerbation [17]. A Chinese study on 1724 tic disorder patients also showed that TNF-α among other cytokines increased in levels as symptoms get more severe, although statistical significance was not reached [18].

The above-mentioned studies as well as ours suggest that TNF-α plays a significant role in the etiology of tic disorder. The results of previous studies vary widely. In fact, a recent meta-analysis failed to identify specific cytokines that are significantly associated with Tourette disorder [4]. More comprehensive research on large datasets is necessary to eliminate possible confounding factors and to identify reliable neuroinflammatory biomarkers for tic disorders.

The fact that only patients with mild tic symptoms showed significant results is probably the most important finding of this study. This is not the first study to demonstrate that patient subgroups with different symptom severities exhibit different cytokine profiles. A previous study by Parker-Athill observed patients during the symptom exacerbation and remission phases [17]. Higher TNF-α levels were associated with antipsychotic use during exacerbation phases while increased IL-4 levels were associated with antipsychotic and antibiotic use during remission phases. Another recent study also divided tic disorder patients into minimal, mild, and moderate-to-severe symptom groups, where only the mild symptom group showed increased TNF-α levels [33]. Tao et al. also pointed out that patients with mild symptoms (YGTSS scores below 10) IL-4, IL-10, and IFN-γ levels were significantly lower in medicated patients than in unmedicated patients whereas moderate and severe symptomatic patients did not show differences [18].

These results raise the following fundamental question: Do cytokine levels change with the progression of tic disorders? Tic disorders typically wax and wane in symptom severity over time. Researchers have suggested that severe symptoms are associated with increased neuroinflammatory reactions. Our results suggest that this phenomenon might not be simple, as correlations between cytokine levels and symptom severity were more relevant during the mild or remission phases. Neuro-inflammatory reactions could have “brewed up” during remission phases and quickly reach a stable state as symptoms still developed, thus resulting in no significant correlations during the exacerbation phase. In addition, it is imperative to note that the mild and above moderate tic groups not only significantly differ in the severity of symptoms but also in age and the duration until visit. The fact that the mild tic group is younger, and that the above moderate group has persisted a longer period without treatment might also elucidate the disparities in their cytokine profiles. Future studies on the early exacerbation phases of tic disorders could shed light on possible neuroinflammatory biomarkers of the disease.

Investigating the neuroinflammatory perspective of tic disorders, it was postulated that the temporal duration of experienced inflammation without medical interventions might be of significance. This length of time could have influenced attributes like reactivity of cytokine profiles, thereby potentially modulating the manifestation and progression of the disorder. Unfortunately, despite our investigation, our study did not yield evidence to substantiate this hypothesis.

Our study had a few limitations. First, only 66 participants were included. A larger sample size would have provided more reliable results. Second, we attempted to gather information about the initial onset of tic symptoms to calculate the duration of tic disorder; however, most patients and guardians could not remember the exact time and provided only approximate answers on the year the symptoms emerged. This is probably because most tic symptoms start in a subtle manner, and patients are usually too young to remember the exact moment. We used this information for our analysis, but the results were limited and inaccurate. More precise data on the initial onset and recent aggravation of the disease are necessary in future studies to identify the exact exacerbation and remission phases. Third, our samples were taken throughout the course of tic disorder, but it remains ambiguous whether the observed changes are a precursor or a result of the disorder’s manifestation. Future cohort studies regarding the onset of the disorder would potentially provide further insight to this issue. Fourth, we chose to analyze cytokines that repeatedly demonstrated significant results in previous studies and selected a kit that could investigate as many of these as possible, but it is undeniable that financial constraints were presents during this decision-making process. We hope that future research will allow to analyze a broader array of cytokines, thereby yielding more comprehensive results than those obtained in the present study.

## Conclusions

This study analyzed the possible associations between various cytokine levels and tic disorders, and their correlation with the severity of tic symptoms. Previous studies have concluded that neuroinflammatory pathways play a critical role in tic disorders. Our results suggest IL-1β and TNF-α among other cytokines as possible candidates, but the identification of key elements and research on how they manifest pathological symptoms is yet to be done. Consequently, to advance further, longitudinal cohort studies along with epigenetic or neuroimaging studies seem to be necessary. We highlight the need for more systematic research with the aid of deep learning or artificial intelligence to analyze much larger datasets and clarify the pathogenesis of tic disorders.

## Data Availability

Please contact the corresponding author for data and material.

## Abbreviations

ADHD: Attention deficit hyperactivity disorder
OCD: Obsessive compulsive disorder
PANDAS: Pediatric autoimmune neuropsychiatric disorders associated with streptococcus
PANS: Pediatric acute-onset neuropsychiatric syndrome
TNF: Tumor necrosis factor
IL: Interleukin
K-WISC: Korean version of the Wechsler Intelligence Scale for Children
YGTSS: Yale Global Tic Severity Scale
ANOVA: One-way analysis of variance

## References

[1] Knight T, Steeves T, Day L, Lowerison M, Jette N, Pringsheim T (2012). Prevalence of tic disorders: a systematic review and meta-analysis. Pediatr Neurol.

[2] Joung YS, Lee MS (2021). The therapeutic approaches in children and adolescent with Tourette’s disorder. Precis Future Med.

[3] Swedo SE, Leonard HL, Garvey M, Mittleman B, Allen AJ, Perlmutter S (1998). Pediatric autoimmune Neuropsychiatric Disorders associated with streptococcal Infections: clinical description of the first 50 cases. Am J Psychiatry.

[4] Lamothe H, Tamouza R, Hartmann A, Mallet L (2021). Immunity and Gilles De La Tourette syndrome: a systematic review and meta-analysis of evidence for immune implications in Tourette syndrome. Eur J Neurol.

[5] Dimitrov DS, Voynov V, Caravella JA (2012). Therapeutic proteins. Therapeutic proteins: methods and protocols.

[6] Lee H, Song M, Lee J, Kim JB, Lee MS (2020). Prospective study on cytokine levels in medication-naïve adolescents with first-episode major depressive disorder. J Affect Disord.

[7] Gabbay V, Klein RG, Alonso CM, Babb JS, Nishawala M, De Jesus G (2009). Immune system dysregulation in adolescent major depressive disorder. J Affect Disord.

[8] D’Acunto G, Nageye F, Zhang J, Masi G, Cortese S (2019). Inflammatory cytokines in children and adolescents with depressive disorders: a systematic review and Meta-analysis. J Child Adolesc Psychopharmacol.

[9] Chen MH, Kao ZK, Chang WC, Tu PC, Hsu JW, Huang KL (2020). Increased proinflammatory cytokines, executive dysfunction, and reduced Gray Matter volumes in first-episode bipolar disorder and major depressive disorder. J Affect Disord.

[10] Çolak Sivri R, Bilgiç A, Kılınç İ (2018). Cytokine, chemokine and BDNF levels in medication-free pediatric patients with obsessive-compulsive disorder. Eur Child Adolesc Psychiatry.

[11] Martino D, Zis P, Buttiglione M (2015). The role of immune mechanisms in Tourette syndrome. Brain Res.

[12] Elamin I, Edwards MJ, Martino D (2013). Immune dysfunction in Tourette syndrome. Behav Neurol.

[13] Caldwell PH, Murphy SB, Butow PN, Craig JC (2004). Clinical trials in children. Lancet.

[14] Leckman JF, Riddle MA, Hardin MT, Ort SI, Swartz KL, Stevenson J (1989). The Yale Global Tic Severity Scale: initial testing of a clinician-rated scale of tic severity. J Am Acad Child Adolesc Psychiatry.

[15] Martino D, Dale RC, Gilbert DL, Giovannoni G, Leckman JF (2009). Immunopathogenic mechanisms in tourette syndrome: a critical review. Mov Disord.

[16] Gabbay V, Coffey BJ, Guttman LE, Gottlieb L, Katz Y, Babb JS (2009). A cytokine study in children and adolescents with Tourette’s disorder. Prog Neuropsychopharmacol Biol Psychiatry.

[17] Parker-Athill EC, Ehrhart J, Tan J, Murphy TK (2015). Cytokine correlations in youth with tic disorders. J Child Adolesc Psychopharmacol.

[18] Tao Y, Xu P, Zhu W, Chen Z, Tao X, Liu J (2022). Changes of cytokines in Children with Tic Disorder. Front Neurol.

[19] Bos-Veneman NG, Bijzet J, Limburg PC, Minderaa RB, Kallenberg CG, Hoekstra PJ (2010). Cytokines and soluble adhesion molecules in children and adolescents with a tic disorder. Prog Neuropsychopharmacol Biol Psychiatry.

[20] Turner MD, Nedjai B, Hurst T, Pennington DJ (2014). Cytokines and chemokines: at the crossroads of cell signalling and inflammatory Disease. Biochim Biophys Acta.

[21] Bloch MH, Leckman JF (2009). Clinical course of Tourette syndrome. J Psychosom Res.

[22] Ho CS, Huang JY, Yang CH, Lin YJ, Huang MY, Su YC (2020). Is the Yale Global Tic Severity Scale a valid tool for parent-reported assessment in the paediatric population? A prospective observational study in Taiwan. BMJ Open.

[23] Sallee F, Kohegyi E, Zhao J, McQuade R, Cox K, Sanchez R (2017). Randomized, Double-Blind, placebo-controlled trial demonstrates the efficacy and safety of oral aripiprazole for the treatment of Tourette’s disorder in children and adolescents. J Child Adolesc Psychopharmacol.

[24] Uzun S, Kozumplik O, Mimica N, Folnegović-Smalc V (2005). Aripiprazole: an overview of a novel antipsychotic. Psychiatria Danubina.

[25] Huang ML, Peer AV, Woestenborghs R, De Coster R, Heykants J, Jansen AA (1993). Pharmacokinetics of the novel antipsychotic agent risperidone and the prolactin response in healthy subjects. Clin Pharmacol Ther.

[26] Wudarsky M, Nicolson R, Hamburger SD, Spechler L, Gochman P, Bedwell J (1999). Elevated prolactin in pediatric patients on typical and atypical antipsychotics. J Child Adolesc Psychopharmacol.

[27] Miller DD, Andreasen NC, O’Leary DS, Rezai K, Watkins GL, Boles Ponto LL (1997). Effect of antipsychotics on regional cerebral blood flow measured with positron emission tomography. Neuropsychopharmacology.

[28] Wirleitner B, Neurauter G, Schröcksnadel K, Frick B, Fuchs D (2003). Interferon-gamma-induced conversion of tryptophan: immunologic and neuropsychiatric aspects. Curr Med Chem.

[29] Raison CL, Miller AH (2003). When not enough is too much: the role of insufficient glucocorticoid signaling in the pathophysiology of stress-related disorders. Am J Psychiatry.

[30] Leckman JF, Katsovich L, Kawikova I, Lin H, Zhang H, Krönig H (2005). Increased serum levels of interleukin-12 and Tumor necrosis factor-alpha in Tourette’s syndrome. Biol Psychiatry.

[31] Li Y, Wang X, Yang H, Li Y, Gui J, Cui Y (2022). Profiles of Proinflammatory cytokines and T cells in patients with Tourette Syndrome: a Meta-analysis. Front Immunol.

[32] Rodríguez N, Morer A, González-Navarro EA, Serra-Pages C, Boloc D, Torres T (2017). Inflammatory dysregulation of monocytes in pediatric patients with obsessive-compulsive disorder. J Neuroinflammation.

[33] Yeon SM, Lee JH, Kang D, Bae H, Lee KY, Jin S (2017). A cytokine study of pediatric Tourette’s disorder without obsessive compulsive disorder. Psychiatry Res.

